# Efflux dynamics of the antiseizure drug, levetiracetam, through the P-glycoprotein channel revealed by advanced comparative molecular simulations

**DOI:** 10.1038/s41598-022-17994-3

**Published:** 2022-08-11

**Authors:** Esmaeil Behmard, Ebrahim Barzegari, Sohrab Najafipour, Amin Kouhpayeh, Younes Ghasemi, Ali A. Asadi-Pooya

**Affiliations:** 1 Epilepsy Research Center, Shiraz University of Medical Sciences, Shiraz, Iran; 2 School of Advanced Technologies in Medicine, Fasa University of Medical Sciences, Fasa, Iran; 3 Medical Biology Research Center, Health Technology Institute, Kermanshah University of Medical Sciences, Kermanshah, Iran; 4 Department of Pharmacology, Faculty of Medicine, Fasa University of Medical Sciences, Fasa, Iran; 5 Pharmaceutical Sciences Research Center, Shiraz University of Medical Sciences, Shiraz, Iran; 6 Shiraz University of Medical Sciences, Shiraz, Iran

**Keywords:** Cheminformatics, Epilepsy, Computational biology and bioinformatics, Drug discovery, Molecular biology, Neuroscience, Structural biology

## Abstract

Understanding the precise mechanistic details of the possible binding and transport of antiseizure medications (ASMs) through the P-glycoprotein (P-gp) efflux pump is essential to find strategies for the treatment of patients with epilepsy resistant to ASMs. In the present work, conventional molecular dynamics, binding free energy calculations, steered molecular dynamics and umbrella sampling were applied to study the interactions of levetiracetam and brivaracetam with P-gp and their possible egress path from the binding site. Comparative results for the control drugs, zosuquidar and verapamil, confirmed their established P-gp inhibitory activity. Brivaracetam, a non-substrate of P-gp, demonstrated stronger static and dynamic interactions with the exporter protein, than levetiracetam. The potential of mean force calculations indicated that the energy barriers through the ligand export were the lowest for levetiracetam, suggesting the drug as a P-gp substrate with facile passage through the transporter channel. Our findings also stressed the contribution of nonpolar interactions with P-gp channel lining as well as with membrane lipid molecules to hamper the ASM efflux by the transmembrane exporter. Appropriate structural engineering of the ASMs is thus recommended to address drug-resistant epilepsy.

## Introduction

Epileptic seizures are uncontrollable in about 30–40% of patients receiving appropriate anti-seizure medications (ASMs)^1–3^. For longer than a century, the epilepsy research community has addressed the burden of refractory epilepsy through special attention to the discovery of new ASMs or developing epilepsy surgery techniques. However, the population of patients with drug-resistant epilepsy has not changed substantially. Epilepsy brain surgery is feasible in a limited proportion of patients with drug-resistant seizures, and success with other introduced methods has been marginal^1–3^. Therefore, investigation to discover new ways and strategies to treat drug-resistant epilepsy seems to be crucial.

Pharmacokinetic and transporter hypotheses are the two most important notions proposed to explain the mechanism of resistance to ASMs in patients with epilepsy^4,5^. A high expression of the membrane transport channel, P-glycoprotein (P-gp), plays a pivotal role in both hypotheses. P-gp has been suggested to prevent its drug substrates from reaching their cellular targets by its efflux pumping function in various tissues, thereby reducing the treatment efficacy.

While P-gp export has been established as an underpinning mechanism for resistance to numerous anticancer drugs^6^, research to verify ASMs as substrates for P-gp in the capillary endothelial cells of the blood brain barrier has led to conflicting findings^4,5,7,8^. Most of the common ASMs are antagonists of ion channels^9^, which propose their possibility to also interact with P-gp. Racetam-derived ASMs, such as levetiracetam and its more potent analog, brivaracetam^10^, act by inhibiting N-type high-voltage-activated calcium channels, and also by binding to synaptic vesicle protein 2A in the brain^9,10^. Levetiracetam, a widely used ASM with a broad therapeutic spectrum against multiple seizure types, has unclear capability to undergo P-gp efflux^4,5,7,8,11^; some studies have suggested the drug as a probable substrate of P-gp^4,5,7,8^.

The inconsistent P-gp transportability profile of ASMs may be attributable to the limitations associated with the experimental methods set up for their investigations^5,7^. In fact, important details on the bio-macromolecular dynamic behavior of the transmembrane channel remain out of reach and obscure when monitored by cell-based assays, animal experiments, or the clinical research. Advanced computational modeling methods such as molecular dynamics (MD) simulations, on the other hand, offer a more illuminative study tool, with high temporal resolution recording of the molecular behavior in full atomic detail. To date, corroboratory molecular simulations of the P-gp transport system have been applied for analyzing the interaction of various anticancer agents/inhibitors with P-gp and the lipid bilayer^12,13^, or for proposing dynamic models of the transmission of anticancer drugs through the channel^14,15^.

In the current study, we integrated multiple enhanced process simulation approaches to study the interactions and transport of the ASMs, levetiracetam and brivaracetam, through the outward-facing conformation of human P-gp (hP-gp) to the extracellular space^16^ (Supplementary Fig. S1 online). To have controls with established and remarkably different transport properties, which could also mark the distinction between P-gp inhibitors and substrates^6^, verapamil, a calcium-channel blocker used for ventricular rate control in various diseases and with proven P-gp inhibitory activity, and zosuquidar, an experimental strong P-gp inhibitor, were comparatively studied^17–19^. We also investigated the role of membrane lipid molecules in the drugs’ dynamics along the TM channel outwards^6^. The results provide insights into the complete translocation pathway and the dynamic mechanisms involved in the possible ASMs’ export by the channel protein, thus helping address the issue of drug-resistance in epilepsies.

## Results

### Stability of the studied systems

**Figure 1:**
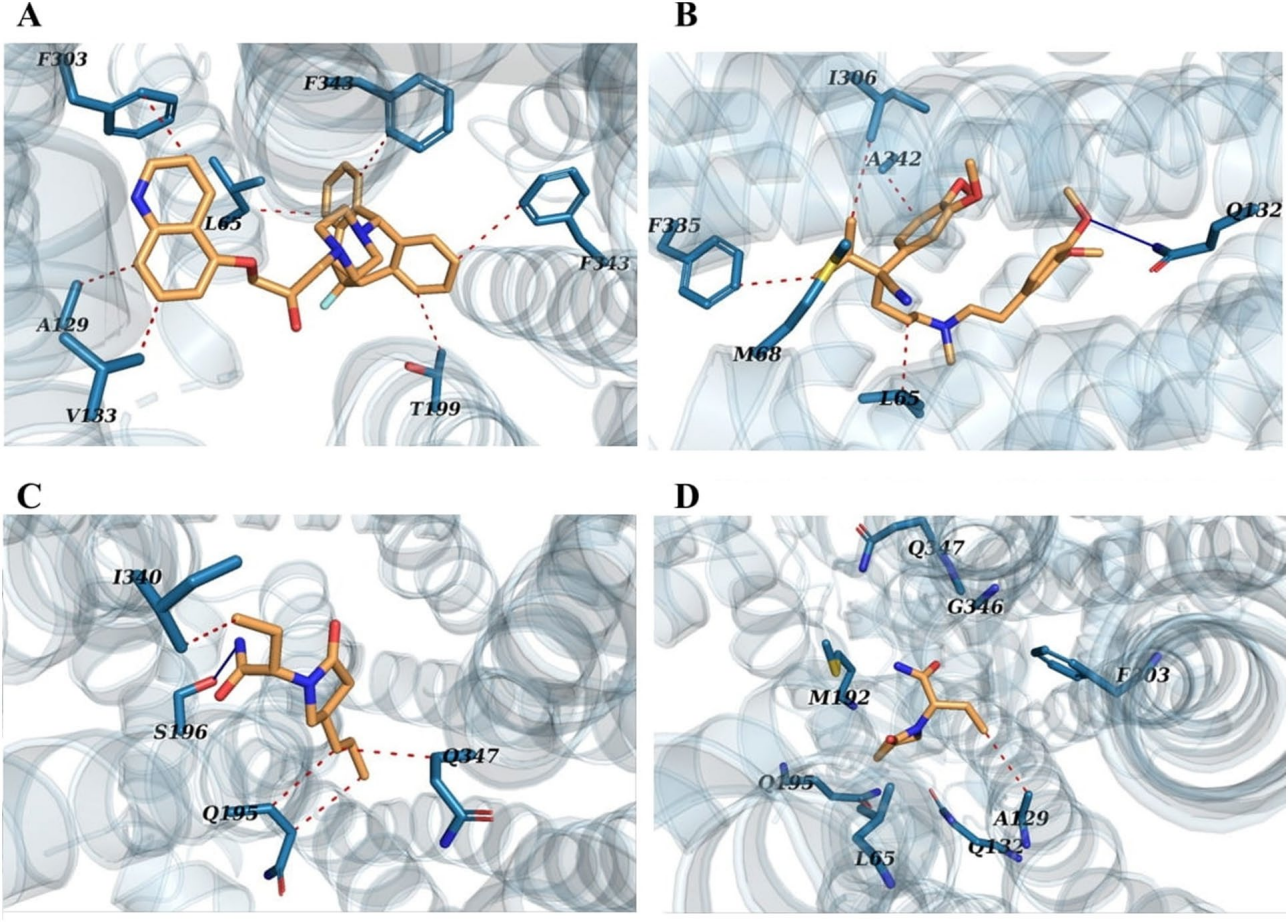
Human P-gp complex with (**A**) zosuquidar, (**B**) verapamil, (**C**) brivaracetam, and (**D**) levetiracetam, after molecular dynamics simulations. hP-gp is represented as light blue cartoon, and ligands as wheat sticks. The key residues (blue sticks) are labeled. Hydrogen bonds are shown as blue line, and hydrophobic interactions in red dots.

To simulate the transport through the hP-gp channel for the ASM, levetiracetam, in comparison with its analogue brivaracetam, the anticancer verapamil, and the P-gp inhibitor zosuquidar, firstly each drug was bound to the efflux protein using the molecular docking approach. Results were indicative of a strong P-gp binding for zosuquidar and verapamil, while the ASMs, particularly levetiracetam, were involved by weak interactions (Tables S1, S2, S3 and S4**)**. The systems then underwent conventional MD simulations to provide stable P-gp-drug complexes for the study of ligand transport from the binding site to the extracellular space using SMD and umbrella sampling methods. Figure 1 displays a zoom of the positions of the ligands in the drug binding site of hP-gp after the protein–ligand complexes were permitted to relax for 100 ns of classical MD. Illustration of the residues involving in the interaction with the highest-affinity binding pose of the ligands in the hP-gp channel indicates that non-polar and aromatic residues played the predominant role in the hP-gp-drugs binding (Fig. 1A–D and Supplementary Fig. S2 online).

The dynamic stability of the protein in the hP-gp-drug complex, as estimated by the root mean squared deviation (RMSD) parameter of the protein Cα atoms, indicated that the protein molecule retained its structural stability throughout the simulations (Supplementary Fig. S3A online). To study the dynamic behavior of the drug molecules at the binding pocket during the simulation time, displacement of ligands’ heavy atoms relative to their initial position was calculated (Supplementary Fig. S3B online). The results showed that the conformation of the ligands at the binding pocket were highly stable and the interactions between the ligands and the residues of the binding site was strong enough to hold the drug molecules in the binding pocket throughout the simulation time (Supplementary Fig. S3B online). These outcomes gave us the assurance that the output was reliable to perform the binding free energy computations respecting the snapshots extracted from the MD trajectory. In addition, the frames produced by MD simulations were reliable enough to contribute to the answer of the questions of ligands’ dynamics along the efflux channel.

### Binding energy profile of the drugs

The estimated binding free energy of the pharmaceutical ligands in their interactions with hP-gp, and the share of different energy components in the binding process are listed in Table 1. Based on these results, the favorable component of the free energy tends to be of non-polar nature. Thus, it can be concluded that hydrophobic interactions are the main driving force of the binding process in the attachment of all studied drugs to hP-gp. Notably, the total free energy and its polar/non-polar contributions indicate a less favorable P-gp binding for levetiracetam and briveracetam in comparison with verapamil and zosuquidar. This is attributable to the smaller size and number of interacting groups on the molecular structure of both ASMs, compared to the P-gp inhibitors (Supplementary Fig. S1 online).

**Table 1:** The computed binding free energy and contribution of the energy elements (kJ/mol).

Energy components	P-gp/Zosuquidar	P-gp/Verapamil	P-gp/Briveracetam	P-gp/Levetiracetam
Electrostatic energy (∆E_ele_)	0.8 ± 0.04	3.1 ± 0.16	1.1 ± 0.05	− 14.5 ± 2.4
van der Waals energy (∆E_vdW_)	− 220.2 ± 13.6	− 225.6 ± 21.9	− 115.5 ± 13.6	− 80.8 ± 2.8
Polar solvation energy (∆G_sol-pol_)	90.4 ± 3.0	109.6 ± 8.7	74.1 ± 2.9	53.9 ± 9.5
Non-polar solvation energy (∆G_sol-np_)	− 22.8 ± 1.4	− 27.0 ± 1.2	− 14.1 ± 0.4	− 11.4 ± 0.49
Polar contribution (∆E_polar_)^a^	91.2 ± 2.0	112.6 ± 4.4	75.2 ± 1.3	39.5 ± 5.6
Non-polar contribution (∆E_non-polar_)^b^	− 243.1 ± 6.8	− 252.6 ± 11.5	− 129.6 ± 7.0	− 92.2 ± 1.6
Binding free energy (∆G_bind_)	− 151.9 ± 4.3	− 139.9 ± 8.0	− 54.4 ± 4.0	− 52.7 ± 3.6

^a^∆E_polar_ = ∆G_sol-pol_ + ∆E_ele_.

^b^∆E_nonpolar_ = ∆G_sol-np_ + ∆E_vdw_.

### Force fluctuations and energy components during SMD simulations

**Figure 2:**
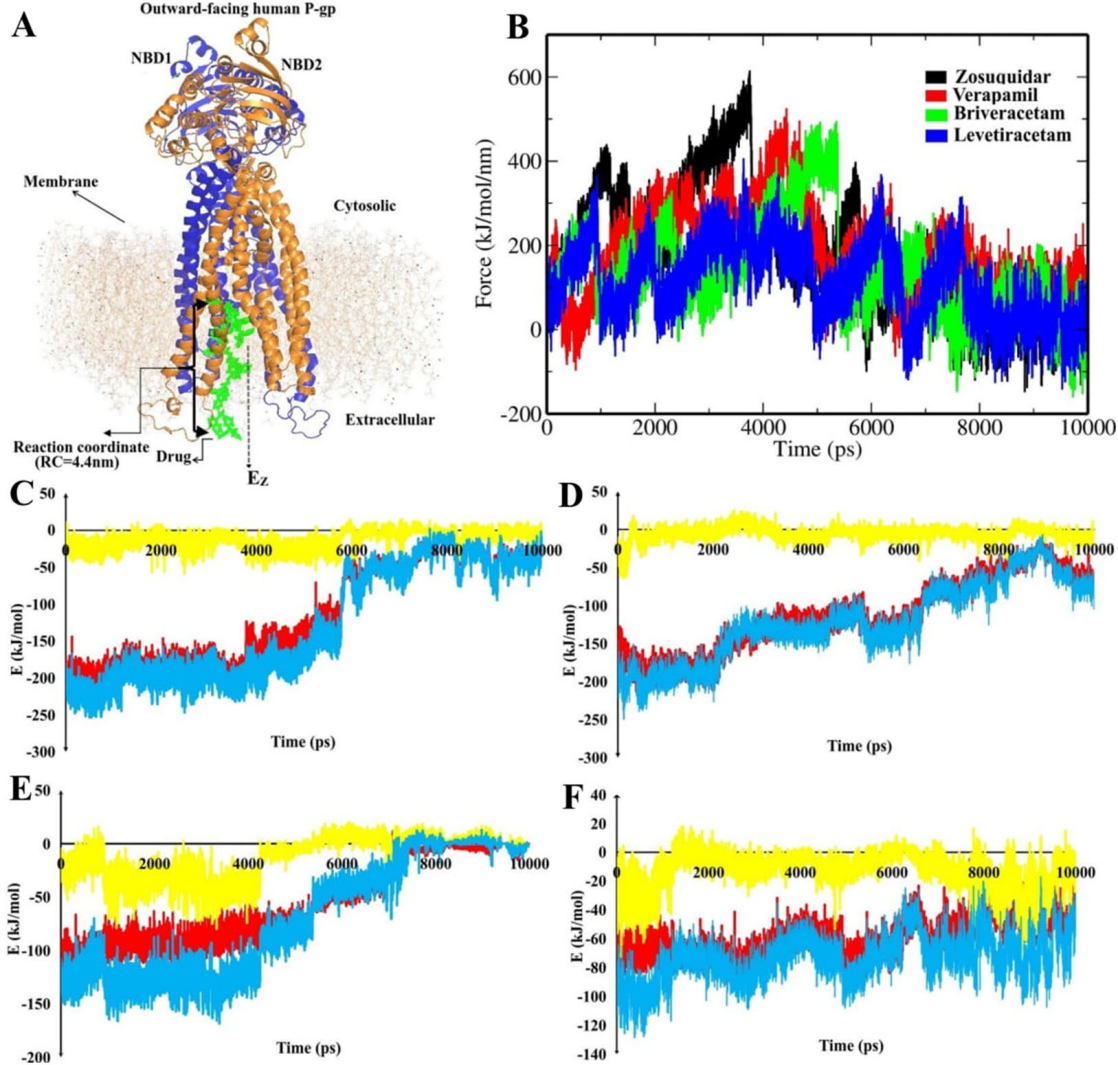
(**A**) Structure of P-gp in the bilayer membrane at the beginning of SMD, and different binding modes of a drug moving along the channel. (**B**) Force changes during the drug transport process through the SMD simulation. (**C**–**F**) Graphs of the SMD energies between P-gp and respectively zosuiquidar, verapamil, briveracetam and levetiracetam, indicating the short-range Coulombic interactions (yellow), short-range Lennard–Jones interactions (red), and the total interaction energy (blue).

The SMD-simulated dynamics of the variation of interactions in the process of drug transport along the P-gp channel are illustrated in Fig. 2. The initial system and the applied reaction coordinate (RC) can be seen in Fig. 2A. The diagram of the force received by ligands moving along the RC during hauler of the drugs from P-gp (Fig. 2B) indicates some local minima in the energy fluctuations in the process of drug’s export, which represent favorable P-gp-drug binding geometries along the channel. Pulling the drugs out along the exporter requires the force to be expanded to break the stabilizing bonds, leading to peaks in the SMD force plot (Fig. 2B).

Binding energies through the SMDs were broken down to short range Coulombic (Coul-SR) and Lennard–Jones (LJ-SR) components to get the electrostatic and vdW contributions, respectively (Fig. 2C–F). The fluctuation patterns of these energies during the SMD were similar to that in the SMD force diagram; i.e. local minima corresponded to larger energy contributions. At the starting points, each drug-P-gp complex is stable with a negative free energy. The energies then decrease and fluctuate during SMD, continuing until all components reach zero, which shows that the drug is completely extruded from the P-gp egress (Fig. 2C–F). Concordantly, the total number of contacts formed between the drugs and hP-gp (Supplementary Fig. S4 online) decreases sharply at about 6000 ps time point of the simulation, for all the drugs. The number of bonds between levetiracetam and the protein is significantly lower than that for the other drugs, and demonstrates a less steep fall with time, which indicates a mild passage and release from the channel.

In all the energy trends, vdW components play the major role in the drug moving process along the P-gp channel (Fig. 2C–F). Thus, hydrophobic interactions may be an important agent in the drug transfer process. The pattern of both total contacts and total interaction energy correspond to the trend of the vdW energies (Fig. 2C–F). This could emphasize the rate-limiting role of the hydrophobic interactions in the drug efflux through the channel. Therefore, drugs that exhibit more nonpolar interactions with hP-gp are expected to encounter larger energy barriers as they cross the channel, making it more difficult for them to transport out of the cell. Comparatively among the studied drugs, zosuquidar and verapamil showed most, and levetiracetam the least hydrophobic interactions with the lining residues along the P-gp channel. This means that compared to other drugs, zosuquidar barely crosses the P-gp exporter, while levetiracetam crosses the channel easily.

### Contribution of lipid molecules to export of ASMs

**Figure 3:**
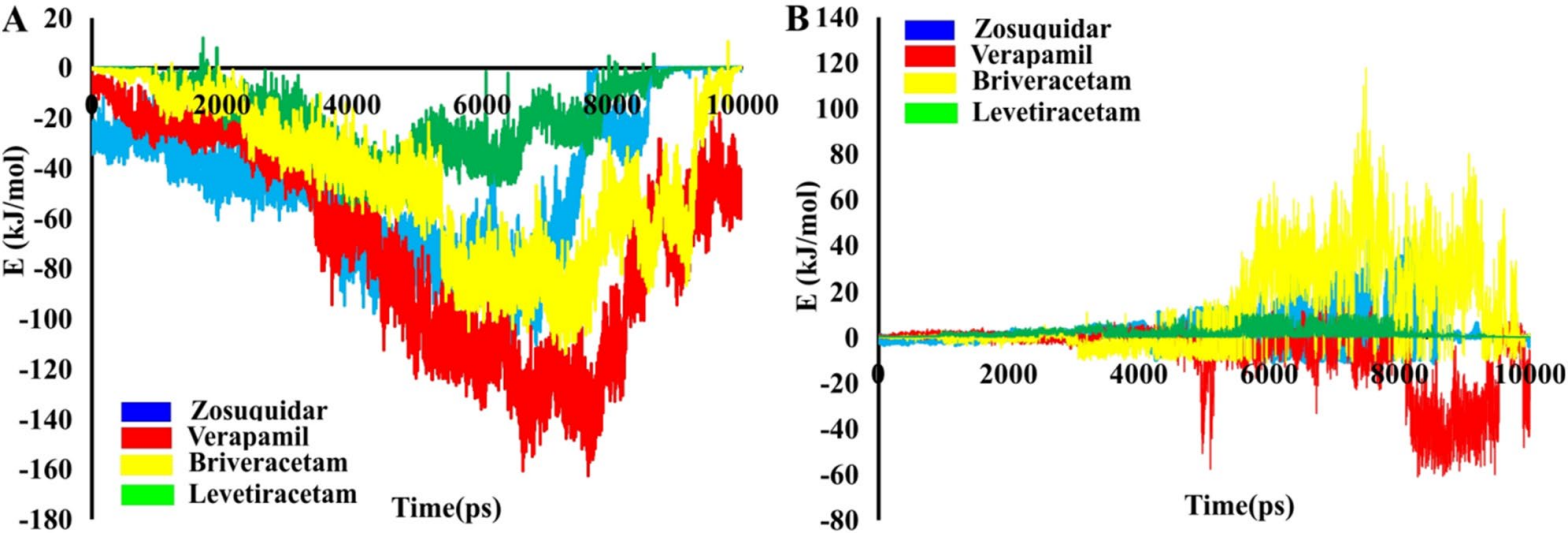
(**A**) van der Waals, and (**B**) electrostatic energies for the interactions of drugs with membrane bilayer lipids.

To clarify the role of lipid molecules of the bilayer membrane in the drugs’ transport process, the interaction energies between the drugs and lipids were analyzed in terms of separate electrostatic and vdW components (Fig. 3). The energetic contributions from lipids can be seen to be comparable with hP-gp protein residues. The vdW component has a much greater share than electrostatics in the interaction with drugs during their transmission through hP-gp. In general, the lipid-verapamil interaction was slightly more favorable than the other drugs. The patterns for lipid-zosuquidar and lipid-brivaracetam interactions were almost identical. Among the drugs, levetiracetam showed the least affinity to bind to lipid molecules. The potency of the drugs’ interaction with lipid molecules correlates well with that for binding to hP-gp, and signifies the difficulty of the transport of the drugs accordingly. It can be said that the combined connections from hP-gp and lipid molecules work together to prevent the transport of the drugs.

### Lining of the passageway for the ASMs

**Figure 4:**
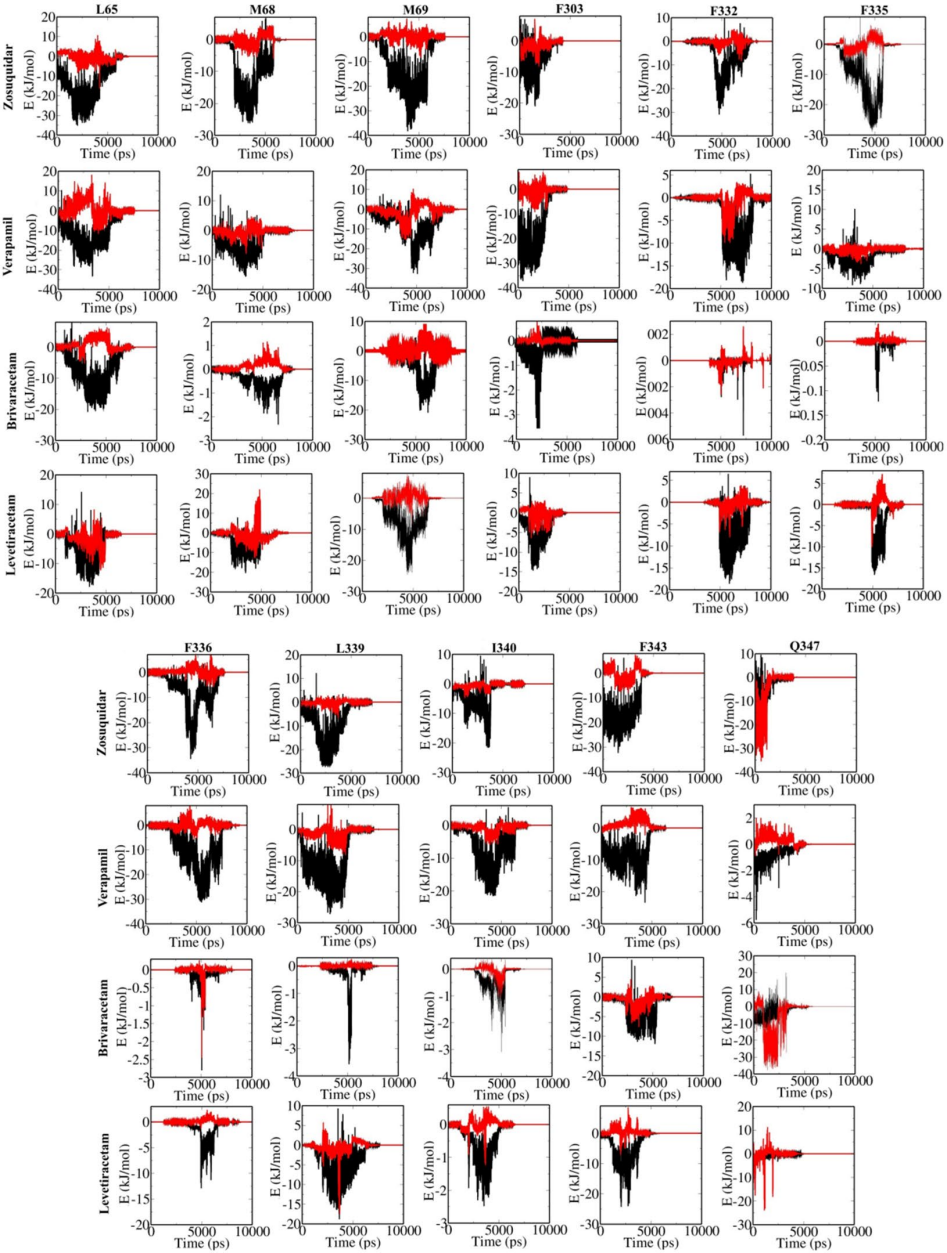
Energy profiles for the key lining residues of the P-gp transport channel in the interaction with studied drugs, showing the van der Waals (black) and electrostatic (red) energy components.

In order to study the roles of residues in the drugs’ moving process along the P-gp channel during the SMD simulation, the dynamic P-gp-drug interactions were analyzed, and revealed a set of mainly hydrophobic residues interacting with the drugs as they passed through the channel (Fig. 4). These included for zosuquidar (L65, M69, F332, F335, F336, F343, Q347), verapamil (L65, M69, F303, F336, L339), brivaracetam (L65, M69, Q347), and levetiracetam (L65, M68, M69, F332, F335, L339, F343). SMD energies for individual lining residues highlighted the role of vdW forces in their interactions with the drugs. Zosuquidar and brivaracetam, both proved as highly potent P-gp binders, indicated also a favorable electrostatic binding to Q347 of P-gp.

Important bindings appear as local and global minima in the energy plots (Fig. 4). The deeper the local minimum a ligand gets stuck in while dynamically interacting with the P-gp transport passageway, the harder it will cross the channel. Thus, deep minima indicate the energy barriers present in the efflux path of the drug. There is an inverse relationship between the ease of drug’s movement in the P-gp channel and the strength of its interaction with lining residues. Our findings show that stronger P-gp-drug interactions in the inner vestibule occur for zosuquidar and verapamil, compared to levetiracetam and brivaracetam. The negative energy values for zosuquidar and verapamil often exceed − 20 kJ/mol, but they are frequently less than − 20 kJ/mol for levetiracetam and even less than − 5 kJ/mol for brivaracetam (though the latter is favored by a strong ionic binding to Q347). These interactions create a larger energy barrier against the passage of the inhibitors through the P-gp channel, while the ASM, levetiracetam, experiences a facile passage along its specific lining in the channel.

### Potential of mean force from umbrella sampling

**Figure 5:**
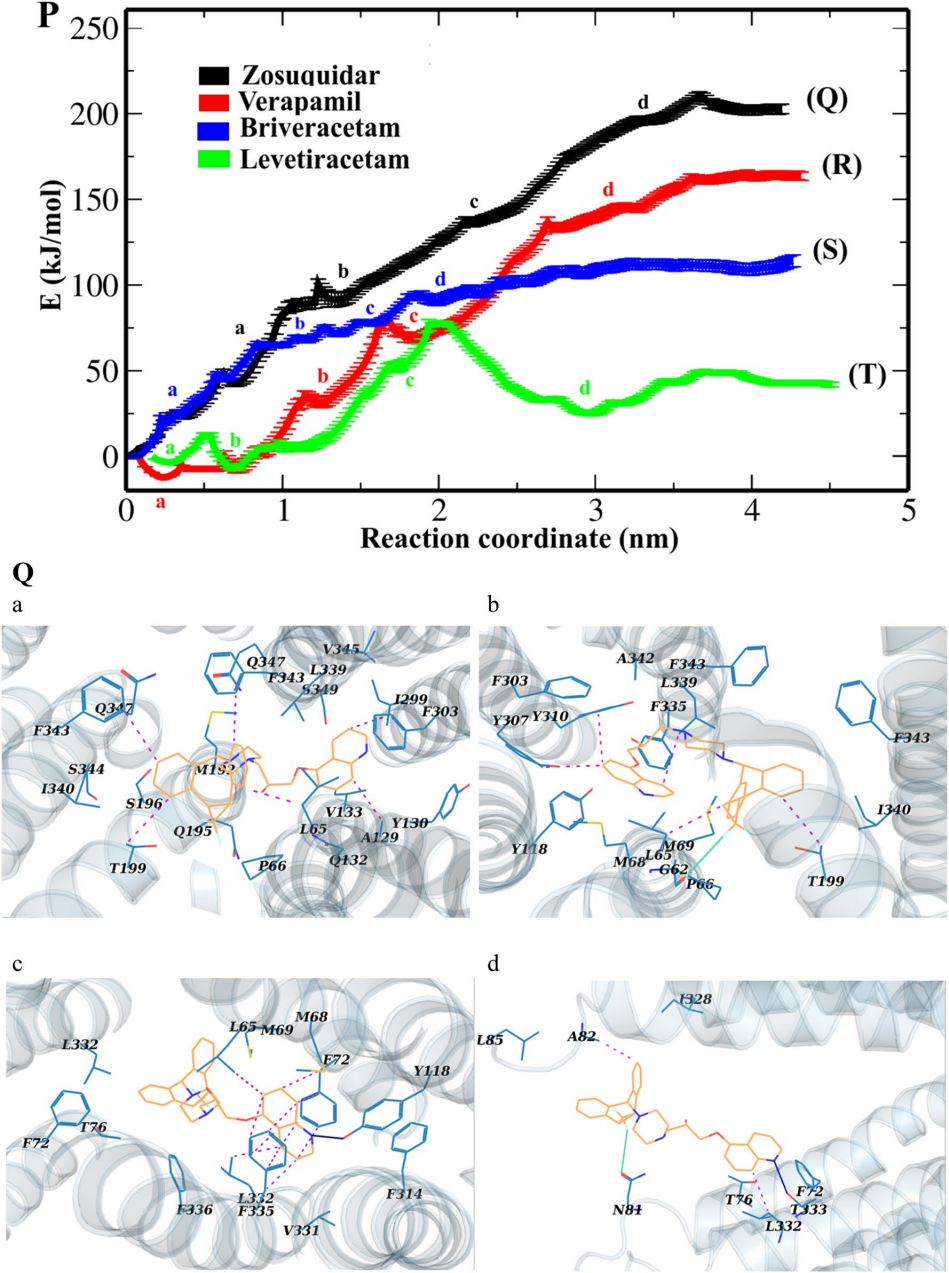

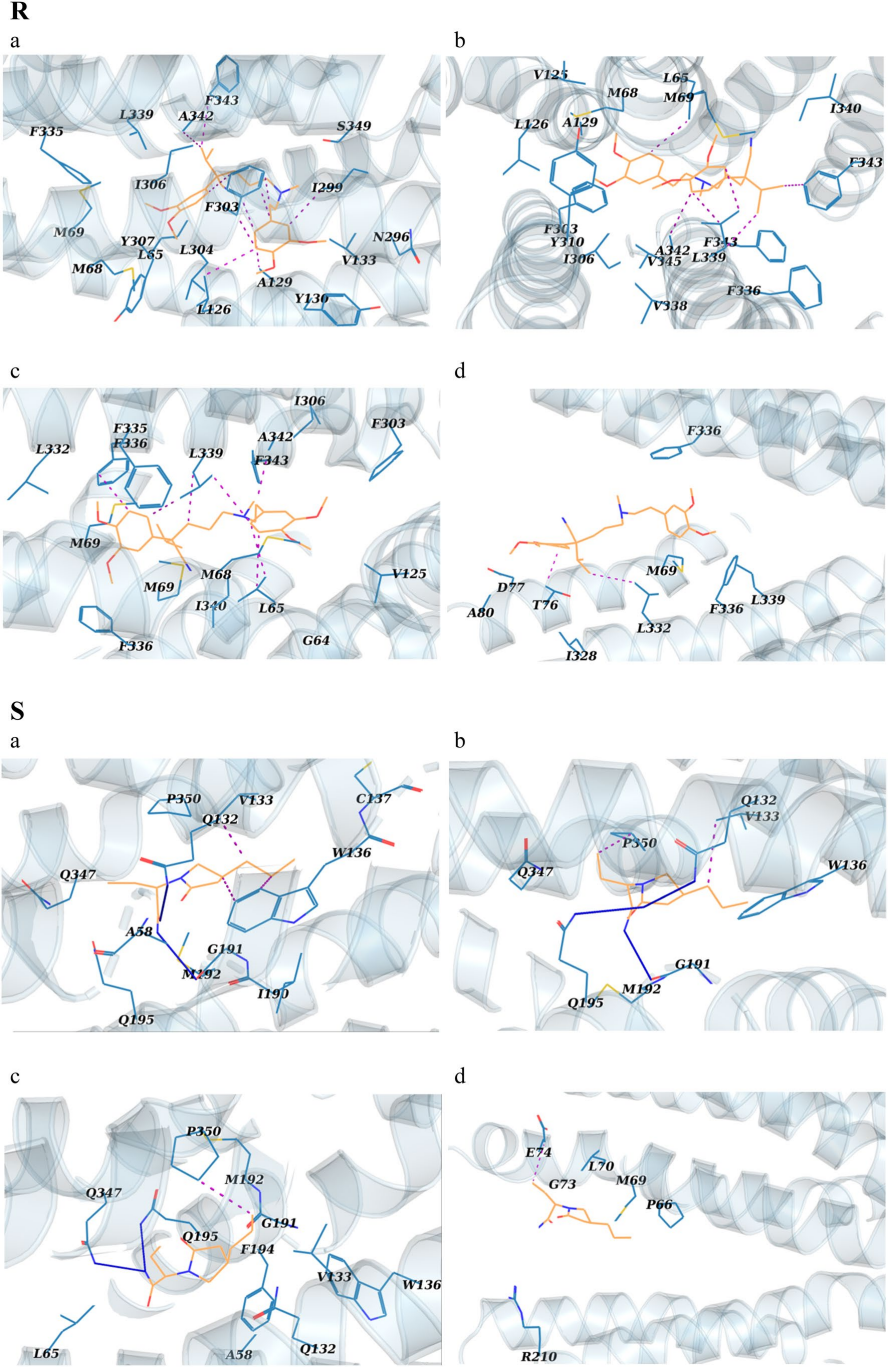

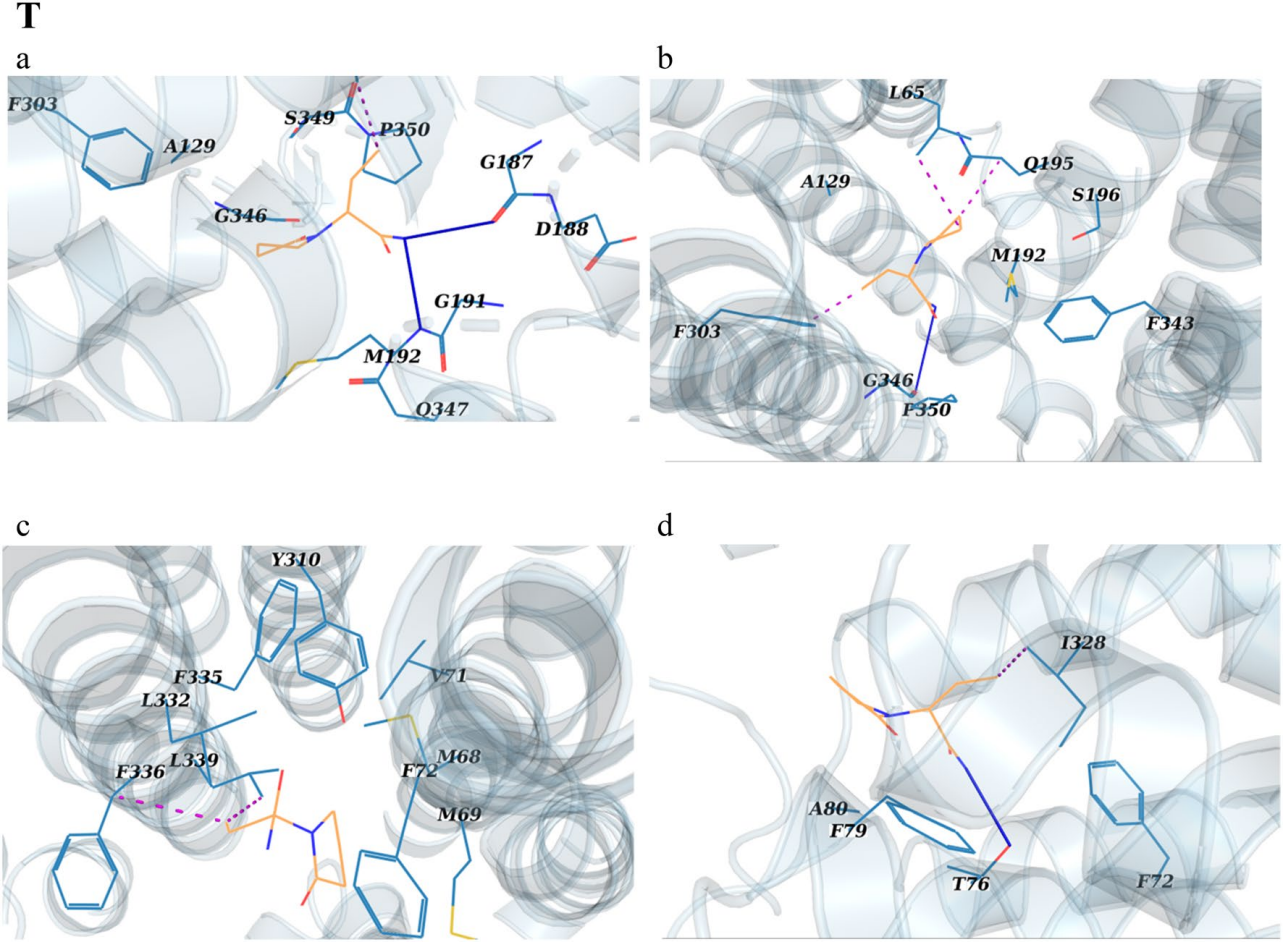
(**P**) The PMF profile change for the transport of zosuquidar, verapamil, brivaracetam and levetiracetam through the hP-gp channel. The a-d tags represent the main intermediate structures of hP-gp, whose snapshots are illustrated in the panels Q–T for the drugs, respectively. (**Q**–**T**) Interactions between the drugs and the inner residues of the main intermediate structures of hP-gp channel during umbrella sampling simulations. Drugs are shown as wheat sticks. The lining residues (blue sticks) are labeled. Hydrogen bonds are shown as blue lines, hydrophobic interactions as red dots, and the halogen bond as a green line.

To gain a deeper understanding of the dynamic drug-residue interactions along the hP-gp channel, the energy fluctuations relevant to drugs’ transmembrane export during SMD were calculated through the umbrella sampling technique (Fig. 5). The computed potential of mean force (PMF) of drugs through the channel is presented in Fig. 5P, and structurally analyzed in the associated panels for zosuquidar (Fig. 5Q), verapamil (Fig. 5R), brivaracetam (Fig. 5S), and levetiracetam (Fig. 5T).

According to Fig. 5Q(a–d), when zosuquidar moves along the P-gp channel, various hydrophobic interactions and a halogen bond occur between the drug and the lining residues. As a result, the PMF diagram increases steadily with a sharp slope (0.3–3.66 nm of RC; Fig. 5P), then achieves to a plateau at 3.66 nm (Fig. 5P), meaning that the compound is completely extruded from the channel. A maximum energy barrier of about 210 kJ/mol is encountered along the drug’s passage through the inner vestibule of the exporter. At the beginning of the transport process, the PMF diagram of verapamil reduces from 0 to − 12.27 kJ/mol, then the molecule is trapped into its main minimum at RC ≈ 0.25 nm (Fig. 5P,R a). To break this barrier, the drug must ascend to a ≈ 92.12 kJ/mol of energy level (Fig. 5P,R a–b). At this point, the system first breaks up the hydrophobic interactions between verapamil and the involving lining residues (L65, L126, A129, I299, F303, L339, A342 and F343) (Fig. 5R a–b); thus, PMF value increases relatively sharply until RC of 1.67 nm. Then, the trend becomes smooth (Fig. 5P,R c–d). At the RC of 3.62 nm, the PMF of verapamil reaches a plateau, meaning that the drug is entirely detached from the P-gp protein (Fig. 5P).

In the diagram for brivaracetam, two different phases can be seen during the transport process (Fig. 5P). A rapid increase phase from 0.1 to 0.84 nm (Fig. 5P,S a ), and a steady slower increase phase from 0.84 to 2.7 nm (Fig. 5P,S b–d). In the rapid increase phase of brivaracetam release, the hydrophobic interactions between the drug and some residues (V133, W136, and I190) are broken (Fig. 5S a); as a result, the value of the PMF increases rapidly (Fig. 5P). The next phase represents the breakage of bonds between the residues E74, V133, Q132, G191, Q132, Q195, Q347 and P350 of the P-gp channel, and brivaracetam during the umbrella sampling simulation time (Fig. 5P and Fig. 5S b–d). Thus, the PMF value gradually increases between points 0.84 and 2.7 nm (Fig. 5P). The PMF diagram of brivaracetam reached plateaus, suggesting the entirely release of the drug from the P-gp transporter protein (Fig. 5P).

It can be seen from the PMF diagram of levetiracetam that the energy is gradually increased from −3.13 to the barrier of 12.12 kJ/mol (Fig. 5P,T a–b), after which it falls into its global minimum (Fig. 5P,T b; RC ≈ 0.69 nm). Obviously, there are hydrophobic and electrostatic interactions between levetiracetam and lining residues in this area (Fig. 5T b). Therefore, the inner vestibule of the P-gp channel has the highest potency to capture levetiracetam; hence, we suggest that this area is the binding site of levetiracetam in the hP-gp channel (Fig. 5T b). According to the PMF diagram, levetiracetam encounters an energy barrier of about 83.2 kJ/mol while crossing the channel (Fig. 5P, RC ≈ 2 nm, and 5T b–d). Therefore, at the beginning of the transport process, the PMF trend of levetiracetam rises rapidly to pass the energy barrier (Fig. 5P, T b–d, RC ≈ 0.69–2 nm). After the drug passes through this area (RC ≈ 2 nm), the diagram gradually reaches plateaus, meaning that the drug has moved away and extruded from the channel (Fig. 5P).

## Discussion

More than one-third of patients with epilepsy demonstrate resistance to ASMs. Failing to assimilate the drug in the intestine or reduced drug uptake at the blood–brain barrier could limit the therapeutic response to drugs. Hypothetically, drug transport through efflux transporters, such as P-gp, plays a significant role in this drug resistance. In the present work, MD and SMD followed by umbrella sampling methods were applied to study the transport behaviors of the ASMs, levetiracetam and brivaracetam, comparatively with zosuquidar and verapamil, along the human P-gp channel. Initially, molecular dockings revealed the lower affinity of levetiracetam for binding to P-gp, than other studied compounds, which suggested it as a P-gp substrate, because the rate of drug transport increases exponentially with decreasing affinity to the transporter^17^. The results of advanced simulations also clearly showed that levetiracetam could be easily transported out of the cytosol through the hP-gp channel. These findings strongly resonate the previous clinical observations that P-gp function may play a significant role in drug-resistant epilepsy^4,5,7,8^.

According to SMD PMFs, the free energy barrier for the transfer of zosuquidar, known as a specific hP-gp inhibitor, from the hP-gp channel was shown to be much larger than that for verapamil, a P-gp antagonist anticancer; thus, the transfer of the former through the channel is much more difficult than the latter. This result is in strong agreement with experimental observations, showing zosuquidar (∆G_unbind_ ≈ − 210 kJ/mol) as a more effective P-gp inhibitor than verapamil (∆G_unbind_ ≈ − 152.36 kJ/mol)^18,19^. In like manner, the PMF energy barriers for levetiracetam passing from P-gp were significantly lower than that of the other drugs, showing a readily export of the ASM drug by the channel. As a result, levetiracetam can be the substrate of hP-gp, which has been suggested in some experimental studies^4,5,7,8^. Additionally, the higher free energy barriers of brivaracetam than levetiracetam suggests that this ASM ligand may transport more difficultly by the channel. Practical research shows that brivaracetam is not a substrate of hP-gp^20^; thus, our computational results are consistent with experimental observations.

Noteworthily, the role of lipid molecules from the bilayer membrane was incorporated in the study of the drug export by P-gp, in addition to the contribution of the channel lining residues^6^. Hydrophobic interactions of the drugs with the inner vestibule regions of P-gp and the membrane lipid molecules increased the energy barriers of the drug transport through hP-gp, consequently restricting the drug passing through the channel. Zosuquidar and verapamil demonstrated the most hydrophobic interactions with hP-gp as well as lipid molecules, compared to other drugs. On the contrary, the PMF fluctuations from umbrella sampling revealed that levetiracetam, which had the weakest hydrophobic interactions with hP-gp as well as lipid molecules among the studied drugs, encountered a smaller energy barrier on its passage through hP-gp and was gently transported by the channel protein. Therefore, decreased vdW interactions between the drug and hP-gp as well as lipid molecules is beneficial to the drug transport process. These findings are strongly consistent with experimental observations. As an example, hydrophobic progesterone was shown to bind to, but did not appear to be transported by, P-gp^21^. In fact, despite being a potent steroid transporter, P-gp does transport hydrophilic steroids more readily than hydrophobic ones; as a result, these steroids may not be good P-gp inhibitors, while most lipophilic steroids can act as strong channel antagonists^21^. Another study reported that antifolate derivatives with more nonpolar nature showed more suppressing activity against resistant tumor cells, than those with more polar groups, because those derivatives less tended to be transported by P-gp^22^.

While experiment replications help provide robust results, our methodology did not include simulation repeats; nevertheless, each system underwent various types of simulations consecutively or in parallel, including 100 ns CMDs followed by 10 ns SMDs and 10 ns US dynamics for each of the 20 windows extracted from each complex system. This equals to the total of ~ 1.6 μs time for the simulations, and allows to have reliable results, like in many similar studies.

## Conclusion

The important ASM, levetiracetam, was shown to easily cross the hP-gp channel, when compared to other control drugs. The facile passage of this antiseizure drug through its specific lining in the channel supports its consideration as a P-gp substrate. From the analyses of interaction energies between the drugs and the channel as well as lipid molecules through steered dynamic modeling, it can be hypothesized that vdW energies are the main driving force in hindering the efflux of drugs by the human P-gp transport channel. Based on the correlation between our computational results and previous experimental observations, our study provides insights to cope with the ASM resistance in refractory epilepsies by manipulation of the hydrophobic interactions between the pharmaceutical compound and both P-gp and membrane lipids. Furthermore, it provides a rational theoretical basis to determining whether a compound acts as a substrate or inhibitor in relation to the hP-gp channel.

## Methodology

### Structure preparation and molecular docking of drugs to hP-gp protein

The outward-facing structure of hP-gp was modeled using SWISS-MODEL (https://swissmodel.expasy.org/; Supplementary Fig. S5 online) based on the crystal structure 2ONJ^16^. As the initial ligand structures, atom coordinates for zosuquidar, verapamil, brivaracetam and levetiracetam were retrieved from Drug Bank (https://go.drugbank.com/). Hydrogen atoms were added to the ligand structures using OpenBabel (http://www.cheminfo.org). Preparation of protein and ligands structures for molecular docking was carried out in AutoDockFR program^23^. Molecular docking was performed using AutoDock Vina open-source tool^24^. For each ligand, the pose with the best binding affinity was selected for further study. To produce 3D diagrams of hydrophobic interactions and hydrogen bonds in the optimal complex of ligands with hP-gp, the PLIP web tool and the open-source molecular visualization software PyMOL were utilized^25,26^.

### Molecular dynamics simulation details

All-atom molecular dynamic simulations were carried out by using the software suite Gromacs-2020^27^. CHARMM-GUI web server (http://www.charmm-gui.org/) was employed for constructing a bilayer membrane model composed of 1-palmitoyl-2-oleoyl-sn-glyucero-3-phosphocholine (POPC), and for insertion of the P-gp-drug complexes into the membrane using the replacement method. Then, the system was solvated by adding water molecules to the simulation box. Sodium and chlorine ions were next added to neutralize the system charge and to simulate a physiological salt concentration of 0.15 M NaCl. The energies and dynamics in all simulation systems were defined by CHARMM36 force field^28^. Periodic boundary conditions were applied in all three dimensions^29^. Topological parameters and atomic point charges for the drug molecules were assigned by the CHARMM-GUI web server. Long-range electrostatics were defined using the particle mesh Ewald (PME) method^30^. The cut-off length for both long-range Coulombic bonds and van der Waals (vdW) interactions was set to 1.2 nm. The steepest descent algorithm was applied to perform energy minimizations^31^. To prevent heat shock, the system temperature was gradually increased to reach the desired temperature (310 K). After that, pressure was applied to the system to achieve the proper density for the system. To avoid the system collapse, NVT and NPT ensembles were implemented with restriction applied on lipids, the protein and the drug. Afterwards, the main simulation was performed for 100,000 ps without any restraints.

### Binding free energy calculation

Molecular mechanics Poisson-Boltzmann surface area (MM-PBSA) method as implemented in g_mmpbsa program was applied to calculate the energy components in the hP-gp-ligand binding^32^. In this approach, the binding free energy (ΔG_bind_) of the receptor-ligand complex is calculated as the difference of the complex free energy (ΔG_RL_) and the free energy of the individual initial substances (ΔG_R_ and ΔG_L_):

1 $$\Delta {\text{G}}_{{{\text{bind}}}} = \, \Delta {\text{G}}_{{{\text{RL}}}} {-} \, \Delta {\text{G}}_{{\text{R}}} {-} \, \Delta {\text{G}}_{{\text{L}}}$$

The free energies are obtained from various energy components:

2 $$\Delta {\text{G }} = \, \Delta {\text{E}}_{{{\text{MM}}}} + \, \Delta {\text{G}}_{{{\text{sol}}}}$$

3 $$\Delta {\text{E}}_{{{\text{MM}}}} = \, \Delta {\text{E}}_{{{\text{vdW}}}} + \, \Delta {\text{E}}_{{{\text{ele}}}}$$

4 $$\Delta {\text{G}}_{{{\text{sol}}}} =_{ } \Delta {\text{G}}_{{{\text{sol}} - {\text{pol}}}} + \, \Delta {\text{G}}_{{{\text{sol}} - {\text{np}}}}$$

The sum of the van der Waals (ΔE_vdW_) and electrostatic (ΔE_ele_) energies represent the gas-phase molecular mechanical energy (ΔE_MM_). The solvation free energy (ΔG_sol_) consists of polar (ΔG_sol-pol_) and non-polar (ΔG_sol-np_) terms^32,33^.

### Steered molecular dynamics

Because the process of the transport for small molecules through membrane channel proteins occurs on a time scale of milliseconds or seconds, it cannot be studied by conventional simulation methods. Steered molecular dynamics (SMD) addresses this issue by introducing an imaginary external force to the small molecule to drive it through the channel^12–15,34,35^. In the present study, the final structures of the channel-drug complexes obtained from conventional simulations were used as the initial model for performing SMD. Harmonic potential was applied to pull out the drug from the channel; this potential causes the force exerted to the drug to change according to the nature of the connections between the drug and the protein. For this purpose, using the force constant of 1000 kJ mol^−1^ nm^−2^, the ligands were hauled out from the binding pocket and moved along the channel (Z-axis) at a constant rate of 0.00044 nm/ps for 10,000 ps SMD. Here, dissociation of the drug from the binding site was considered as the reaction coordinate (RC; ~ 4.4 nm). Other parameters for performing SMD were set according to the conventional simulations. Outputs analyses addressed both drug-channel and drug-lipid interactions.

### Umbrella sampling simulation for drug release

Umbrella sampling simulation is a technique to compute the free energy profile of a chemical reaction, calculated as a potential of mean force (PMF)^12–15,34–36^. This approach involves performing simulations in a series of windows that are extracted from the RC considered in the SMD. At the end of the window series simulation, distributions of samples during the trajectory are combined using the weighted histogram analysis (WHAM) method, and finally an accurate PMF curve is calculated along the RC^12–15,34–36^. In this work, the umbrella sampling approach was adopted to calculate the free-energy profile of the ligands along the hP-gp channel. The Z distance along the RC (~ 4.4 nm) was divided into windows every 0.2 nm. Accordingly, the total of 20 windows along the RC were simulated independently, for each ligand. A harmonic umbrella potential with a force constant of 1000 kJ mol^−1^ nm^−2^, was applied along the Z-axis. Parrinello-Rahman barostat and Nosé-hoover thermostat were used to maintain the pressure of the system at 1 atm, and the temperature at 310 K, respectively^27^. Each window was equilibrated for 1000 ps followed by 8000 ps of production run. Finally, the WHAM tool was applied to calculate the PMF based on the obtained umbrella sample^12–15,34–36^.

## Supplementary Information

Supplementary Information.

## Data Availability

No datasets were generated or analysed during the current study. All data generated or analysed during this study are included in this published article and its Supplementary Information files.
