# Supplementary material for: Efflux dynamics of the antiseizure drug, levetiracetam, through the P-glycoprotein channel revealed by advanced comparative molecular simulations
Source: Sci Rep. 2022 Aug 11;12:13674. doi: 10.1038/s41598-022-17994-3 (PMC9372152; doi:10.1038/s41598-022-17994-3)
Supplement: Supplementary file 1 — Supplementary Information. [file 41598_2022_17994_MOESM1_ESM.docx]

**Efflux dynamics of the antiseizure drug, levetiracetam, through the P-glycoprotein channel revealed by advanced comparative molecular simulations**

**Esmaeil Behmard,^a,b^ Ebrahim Barzegari,^c^ Sohrab Najafipour,^b^ Amin Kouhpayeh,^d^ Younes Ghasemi,^e^ Ali A. Asadi-Pooya ^a,f^**

^a^ Epilepsy Research Center, Shiraz University of Medical Sciences, Shiraz, Iran.

^b^ School of Advanced Technologies in Medicine, Fasa University of Medical Sciences, Fasa, Iran.

^c^ Medical Biology Research Center, Health Technology Institute, Kermanshah University of Medical Sciences, Kermanshah, Iran.

^d^ Department of Pharmacology, Faculty of Medicine, Fasa University of Medical Sciences, Fasa, Iran

^e^ Pharmaceutical Sciences Research Center, Shiraz University of Medical Sciences, Shiraz, Iran.

^f^ Jefferson Comprehensive Epilepsy Center, Department of Neurology, Thomas Jefferson University, Philadelphia, USA.

SUPPLEMENTARY INFORMATION

**Table S1:** Molecular docking results for the binding of P-gp to zosuquidar

| **mode** | **affinity(kcal/mol)** | **dist from best mode (rmsd l.b.)** | **dist from best mode(rmsd u.b.)** |
| --- | --- | --- | --- |
| **1** | **-9.2** | **0** | **0** |
| **2** | **-9.1** | **1.098** | **2.020** |
| **3** | **-8.9** | **2.948** | **5.373** |
| **4** | **-8.9** | **1.288** | **1.696** |
| **5** | **-8.7** | **3.491** | **5.978** |
| **6** | **-8.6** | **3.905** | **6.743** |
| **7** | **-8.6** | **2.423** | **5.352** |
| **8** | **-8.5** | **3.590** | **6.013** |
| **9** | **-8.5** | **3.789** | **6.505** |
| **10** | **-8.4** | **2.618** | **6.417** |
| **11** | **-8.2** | **2.948** | **5.893** |
| **12** | **-8.2** | **2.948** | **4.401** |
| **13** | **-8** | **3.270** | **6.050** |
| **14** | **-7.9** | **2.717** | **4.600** |
| **15** | **-7.9** | **2.553** | **4.011** |
| **16** | **-7.8** | **2.519** | **4.767** |
| **17** | **-7.7** | **3.907** | **6.450** |
| **18** | **-7.7** | **3.357** | **5.568** |
| **19** | **-7.7** | **3.663** | **5.919** |
| **20** | **-7.6** | **4.103** | **7.807** |

**Table S2:** Molecular docking results for the binding of P-gp to verapamil

| **mode** | **affinity(kcal/mol)** | **dist from best mode (rmsd l.b.)** | **dist from best mode(rmsd u.b.)** |
| --- | --- | --- | --- |
| **1** | **-6.6** | **0** | **0** |
| **2** | **-6.5** | **1.574** | **2.653** |
| **3** | **-6.4** | **1.688** | **2.308** |
| **4** | **-6.3** | **3.658** | **6.177** |
| **5** | **-6.2** | **1.469** | **5.820** |
| **6** | **-6.1** | **3.391** | **7.203** |
| **7** | **-6.1** | **3.177** | **5.755** |
| **8** | **-6.0** | **2.006** | **2.815** |
| **9** | **-6.0** | **2.595** | **5.806** |
| **10** | **-5.9** | **3.517** | **7.982** |
| **11** | **-5.9** | **2.725** | **4.871** |
| **12** | **-5.9** | **2.419** | **5.981** |
| **13** | **-5.9** | **3.681** | **6.966** |
| **14** | **-5.8** | **2.239** | **6.363** |
| **15** | **-5.8** | **2.948** | **6.476** |
| **16** | **-5.7** | **3.507** | **6.664** |
| **17** | **-5.7** | **3.029** | **6.089** |
| **18** | **-5.7** | **3.211** | **8.306** |
| **19** | **-5.6** | **3.634** | **6.097** |
| **20** | **-5.6** | **2.791** | **5.746** |

**Table S3:** Molecular docking results for the binding of P-gp to brivaracetam

| **mode** | **affinity(kcal/mol)** | **dist from best mode (rmsd l.b.)** | **dist from best mode(rmsd u.b.)** |
| --- | --- | --- | --- |
| **1** | **-5.8** | **0** | **0** |
| **2** | **-5.8** | **2.271** | **4.327** |
| **3** | **-5.6** | **1.672** | **3.422** |
| **4** | **-5.5** | **2.744** | **4.376** |
| **5** | **-5.5** | **3.264** | **4.589** |
| **6** | **-5.4** | **3.110** | **4.355** |
| **7** | **-5.3** | **1.986** | **3.178** |
| **8** | **-5.3** | **2.186** | **4.631** |
| **9** | **-5.0** | **1.694** | **3.651** |
| **10** | **-5.0** | **2.050** | **3.099** |
| **11** | **-5.0** | **2.665** | **3.901** |
| **12** | **-4.9** | **1.914** | **4.746** |
| **13** | **-4.9** | **2.418** | **4.009** |
| **14** | **-4.9** | **1.933** | **4.032** |
| **15** | **-4.8** | **2.145** | **3.360** |
| **16** | **-4.7** | **2.465** | **4.411** |
| **17** | **-4.6** | **2.229** | **3.419** |
| **18** | **-4.5** | **2.184** | **3.825** |
| **19** | **-4.5** | **3.278** | **5.461** |
| **20** | **-4.4** | **2.428** | **3.465** |

**Table S4:** Molecular docking results for the binding of P-gp to levetiracetam

| **mode** | **affinity(kcal/mol)** | **dist from best mode (rmsd l.b.)** | **dist from best mode(rmsd u.b.)** |
| --- | --- | --- | --- |
| **1** | **-5.5** | **0** | **0** |
| **2** | **-5.3** | **1.962** | **4.124** |
| **3** | **-5.2** | **2.066** | **3.913** |
| **4** | **-5.1** | **1.527** | **4.244** |
| **5** | **-5.1** | **2.189** | **3.452** |
| **6** | **-5.0** | **1.653** | **2.455** |
| **7** | **-5.0** | **2.335** | **4.690** |
| **8** | **-5.0** | **2.393** | **2.653** |
| **9** | **-5.0** | **1.882** | **2.463** |
| **10** | **-4.9** | **2.532** | **2.993** |
| **11** | **-4.9** | **2.874** | **4.336** |
| **12** | **-4.9** | **1.596** | **2.552** |
| **13** | **-4.8** | **1.966** | **3.856** |
| **14** | **-4.8** | **1.983** | **2.744** |
| **15** | **-4.8** | **2.327** | **3.387** |
| **16** | **-4.7** | **2.894** | **3.538** |
| **17** | **-4.8** | **2.558** | **4.037** |
| **18** | **-4.8** | **2.732** | **4.912** |
| **19** | **-4.6** | **2.023** | **4.642** |
| **20** | **-4.6** | **2.403** | **3.127** |


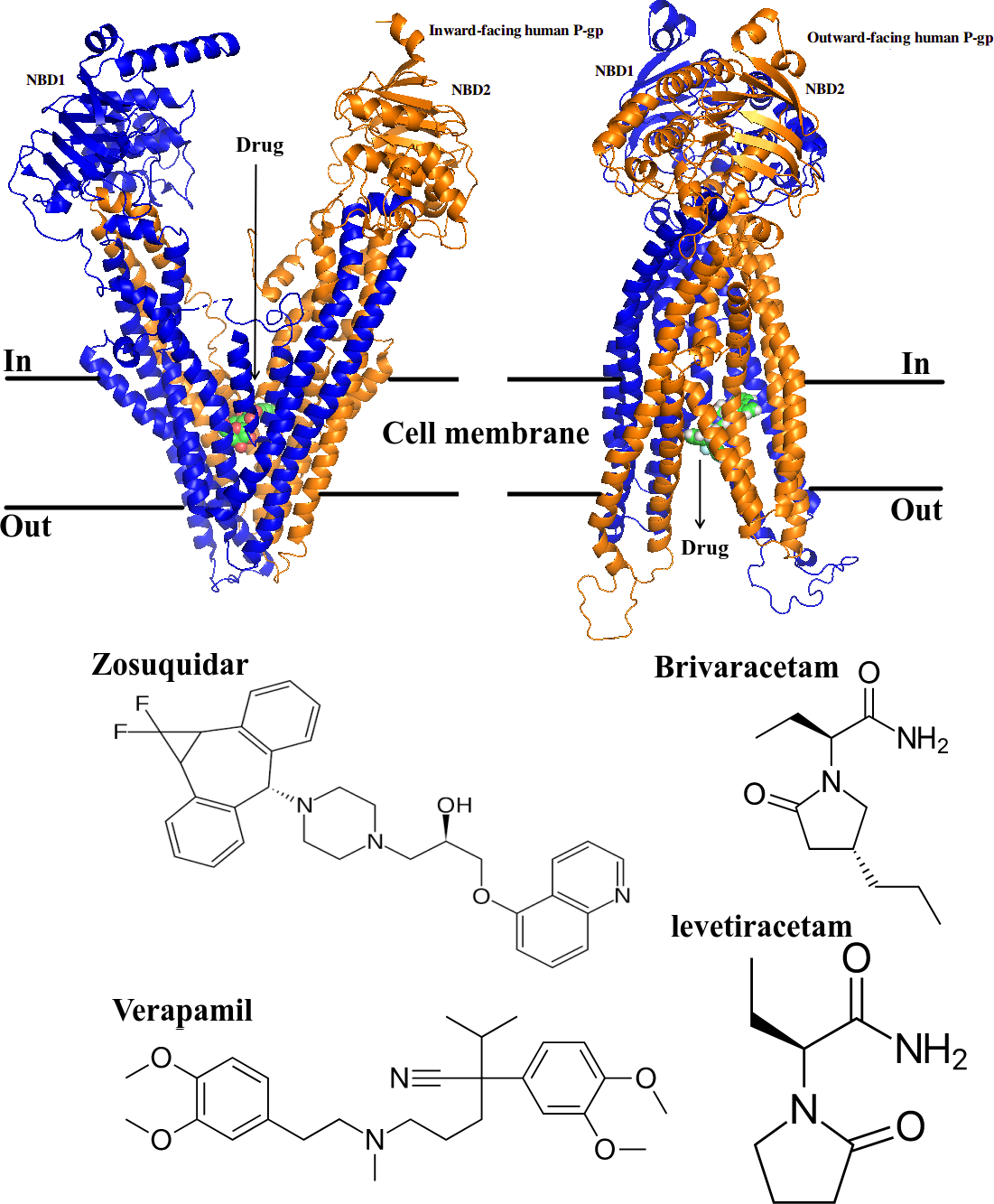


**Figure S1:** (Top) Structural conformation of the P-glycoprotein channel in the inward-facing (PDB ID: 4F4C) and outward-facing states (PDB ID: 2ONJ). Structurally, the export channel is composed of two homologous halves, each comprising a nucleotide-binding domain (NBD) and a transmembrane domain (TM) [1,2]. According to the ATP-switch model, proposed for the drug transfer process by P-gp, the binding of ATP molecules to NBDs results in domain dimerization. A subsequent conformational change in the TM domains first leads to the exposure of the binding site to enable the substrate binding and then progresses from the inward to the outward side to pump the substrate to the out of the cell. The channel finally resumes its inward-facing conformation, by ATP hydrolysis products leaving the NBDs [1,2]. (Bottom) Molecular structure of the studied drugs.

**Figure S2:** Human P-gp complex with (A) zosuquidar , (B) verapamil, (C) brivaracetam, and (D) levetiracetam, after molecular dynamics simulations. ligands are represented as wheat sticks. The binding pocket residues (blue sticks) are labeled. Hydrogen bonds are shown as blue line, and hydrophobic interactions in magenta dots.


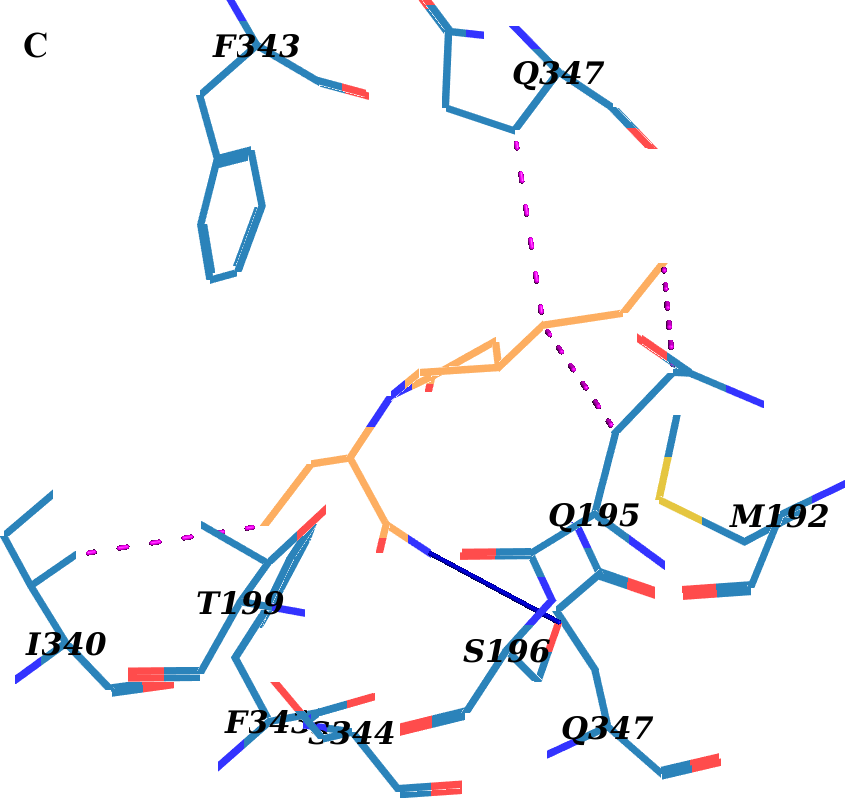

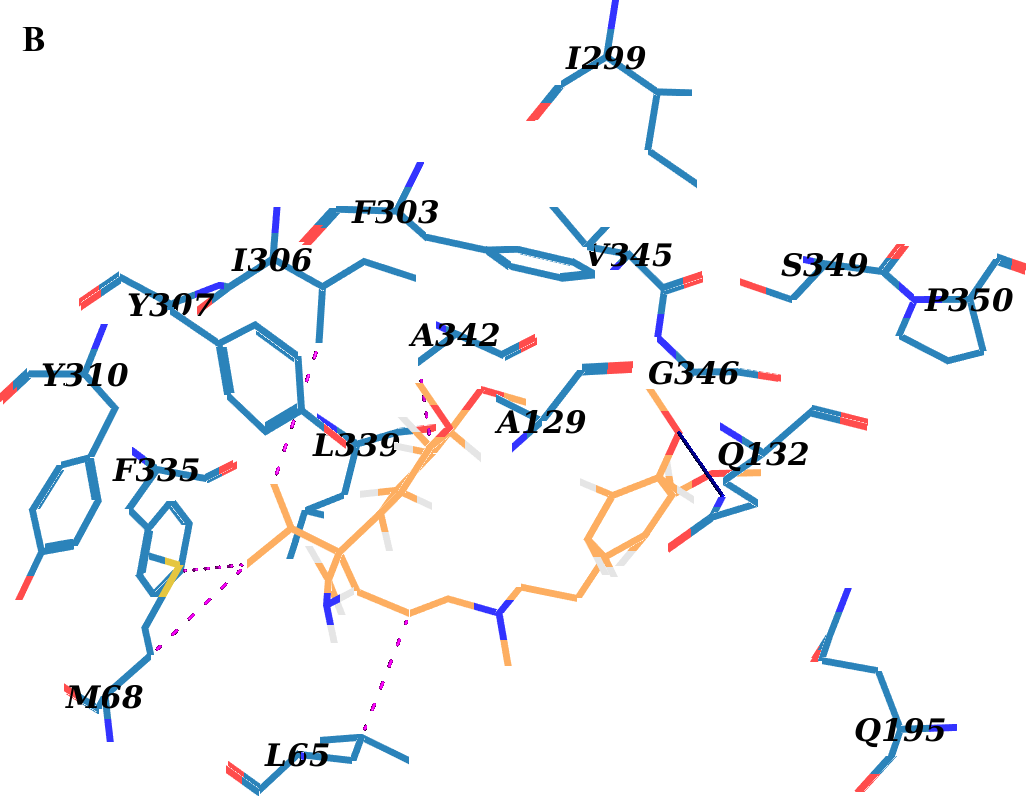

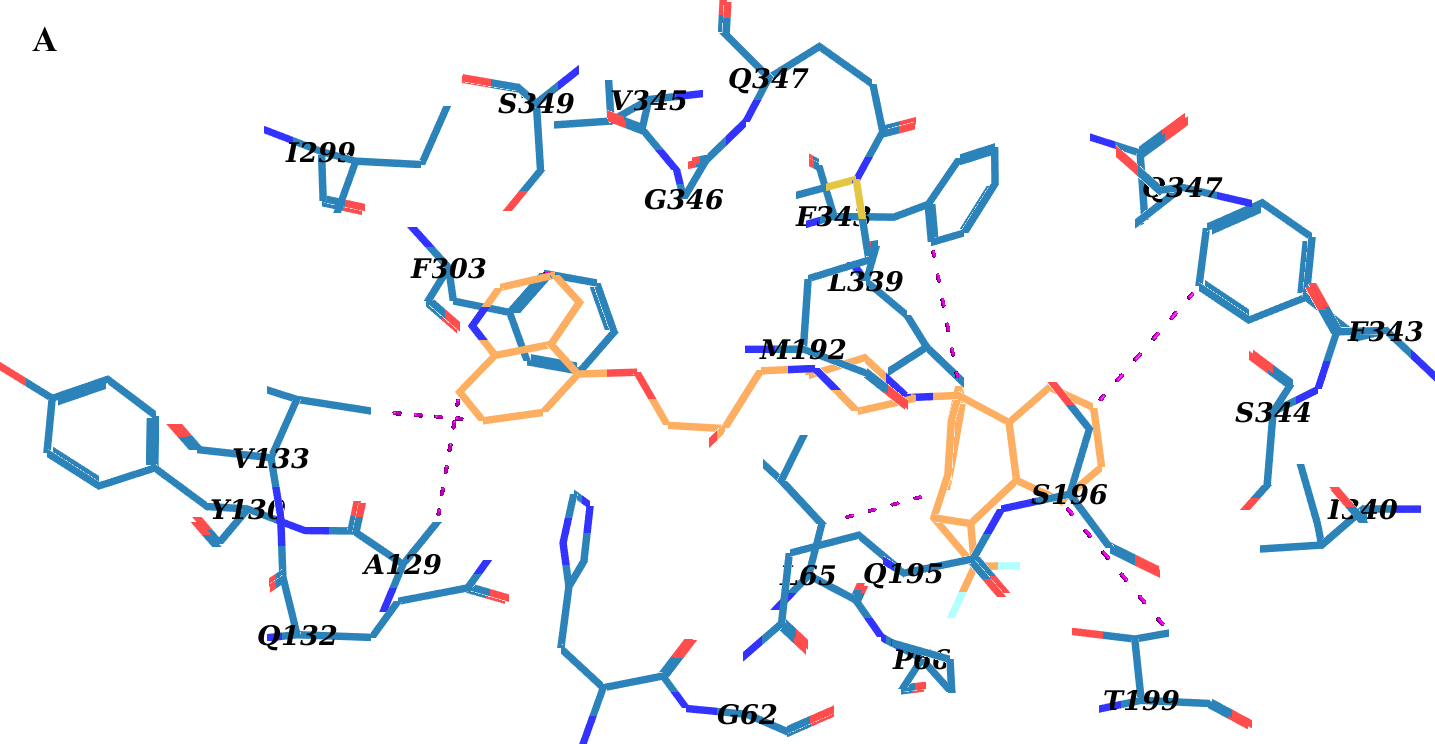

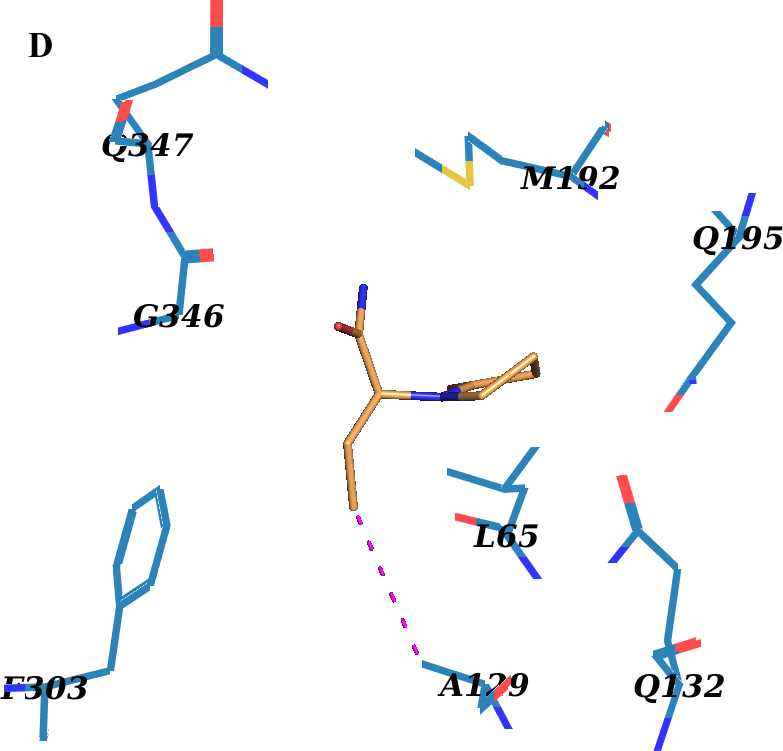


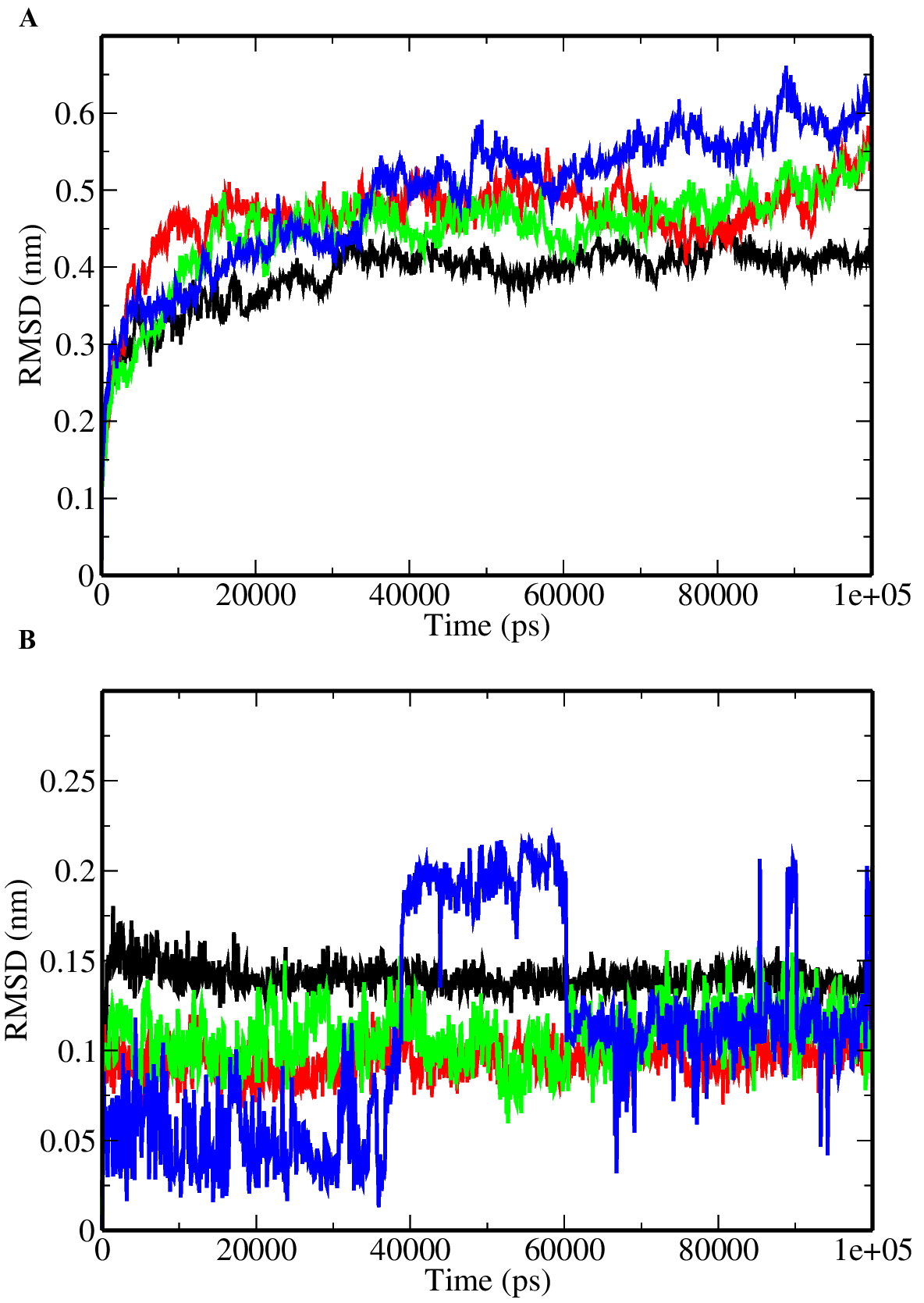


**Figure S3:** Molecular dynamics simulation analyses for complexes of P-gp with the drugs, zosuquidar (black), verapamil (red), brivaracetam (green) and levetiracetam (blue). (A) The Cα-RMSD graph for P-gp; (B) The heavy-atom RMSD of the drug molecules.


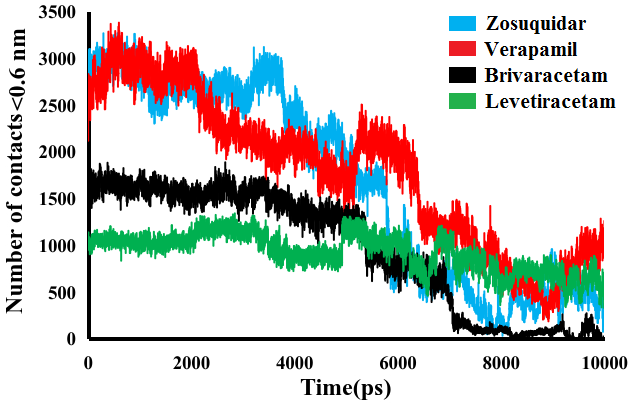


**Figure S4:** The number of contacts between the studied drugs and the hP-gp channel during steered molecular dynamics.


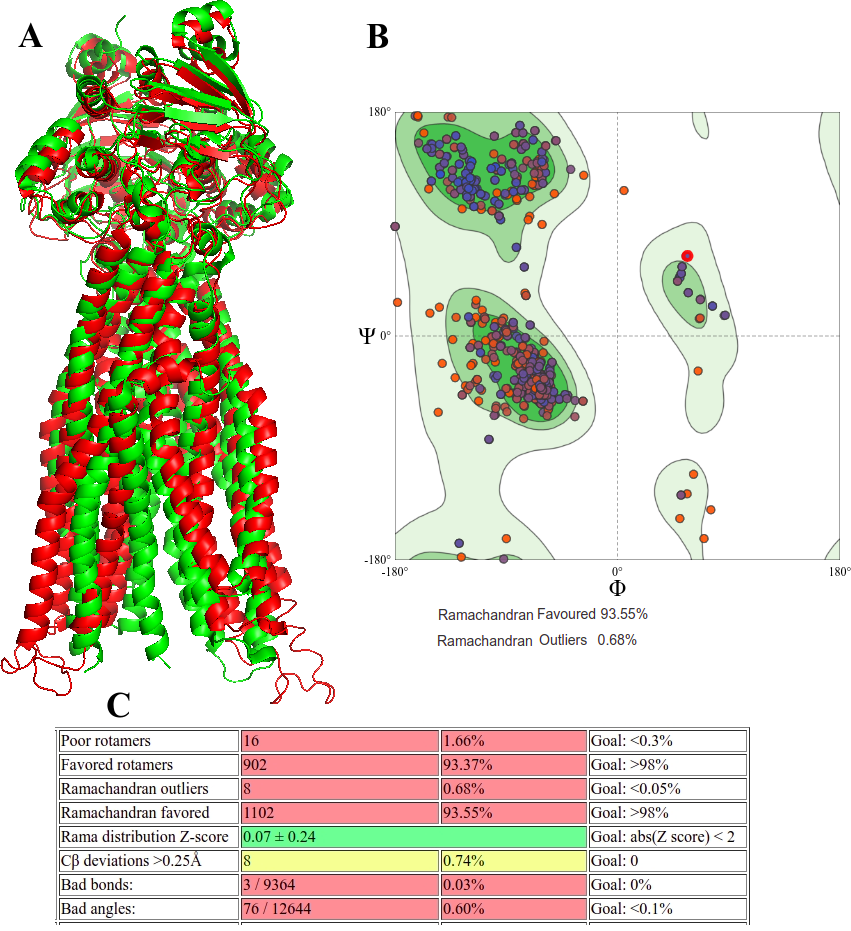


**Figure S5:** Human P-gp (hP-gp) outward-facing structure was modeled based on the crystal outward-facing structure of a bacterial ABC transporter (*S. aureus* Sav1866) [3]. (A) The modeled hP-gp structure (red cartoon), was similar to the recently determined cryo-EM structure of hP-gp (green cartoon; PDB ID: 6C0V; RMSD = 2.6 Å) [4], which was identified using partially opened transmembrane domains. Structure assessment was carried out using SWISS-MODEL (B) and MolProbity server (C), showing high qualityy of the built model.

**References**

1. Higgins, C., Linton, K. The ATP switch model for ABC transporters. Nat Struct Mol Biol 11, 918–926 (2004). [DOI: 10.1038/nsmb836](https://doi.org/10.1038/nsmb836).
2. Urbatsch, I. L., Al-Shawi, M. K., & Senior, A. E. (1994). Characterization of the ATPase activity of purified Chinese hamster P-glycoprotein. Biochemistry, 33(23), 7069-7076. DOI: 10.1021/bi00189a008.
3. Dawson, R. J., & Locher, K. P. (2006). Structure of a bacterial multidrug ABC transporter. Nature, 443(7108), 180-185. DOI: 10.1038/nature05155.
4. Kim, Y., & Chen, J. (2018). Molecular structure of human P-glycoprotein in the ATP-bound, outward-facing conformation. Science, 359(6378), 915-919. DOI: 10.1126/science.aar7389.
